# Rikkunshito Ameliorates Cancer Cachexia Partly through Elevation of Glucarate in Plasma

**DOI:** 10.1155/2015/871832

**Published:** 2015-09-15

**Authors:** Katsuya Ohbuchi, Shin Nishiumi, Naoki Fujitsuka, Tomohisa Hattori, Masahiro Yamamoto, Akio Inui, Takeshi Azuma, Masaru Yoshida

**Affiliations:** ^1^Division of Metabolomics Research, Kobe University Graduate School of Medicine, 7-5-1 Kusunoki-Cho, Chuo-ku, Kobe, Hyogo 650-0017, Japan; ^2^Tsumura Research Laboratories, Tsumura and Co., 3586 Yoshiwara, Ami-machi, Inashiki-gun, Ibaraki 300-1192, Japan; ^3^Division of Gastroenterology, Department of Internal Medicine, Kobe University Graduate School of Medicine, 7-5-1 Kusunoki-Cho, Chuo-ku, Kobe, Hyogo 650-0017, Japan; ^4^Department of Psychosomatic Internal Medicine, Kagoshima University Graduate School of Medical and Dental Sciences, 8-35-1 Sakuragaoka, Kagoshima 890-8520, Japan

## Abstract

Cancer cachexia, which is characterized by decreased food intake, weight loss and systemic inflammation, increases patient's morbidity and mortality. We previously showed that rikkunshito (RKT), a Japanese traditional herbal medicine (Kampo), ameliorated the symptoms of cancer cachexia through ghrelin signaling-dependent and independent pathways. To investigate other mechanisms of RKT action in cancer cachexia, we performed metabolome analysis of plasma in a rat model bearing the Yoshida AH-130 hepatoma. A total of 110 metabolites were detected in plasma and RKT treatment significantly altered levels of 23 of those metabolites in cachexia model rats. Among them, glucarate, which is known to have anticarcinogenic activity through detoxification of carcinogens via inhibition of *β*-glucuronidase, was increased in plasma following administration of RKT. In our AH-130 ascites-induced cachexia rat model, administration of glucarate delayed onset of weight loss, improved muscle atrophy, and reduced ascites content. Additionally, glucarate reduced levels of plasma interferon-*γ* (IFN-*γ*) in tumor-bearing rats and was also found to suppress LPS-induced IFN-*γ* expression in splenocytes *in vitro*. These results suggest that glucarate has anti-inflammatory activity via a direct effect on immune host cells and suggest that RKT may also ameliorate inflammation partly through the elevation of glucarate in plasma.

## 1. Introduction

Cancer cachexia is characterized by anorexia, muscle tissue wasting, and systemic inflammation. It is observed in the majority of terminal cancer patients and is responsible for the death of at least 20% of all cancer patients [1]. Cachexia leads not only to the impairment of quality of life (QOL) but also to reduced tolerance to chemotherapy and radiotherapy [2]. Consequently, cachexia is an important cause of morbidity and mortality [3].

The pathophysiology of cachexia is multifactorial. Although anorexia is frequently associated with the presence of cachexia, anorexia itself does not explain the severe weight loss in cancer cachexia. Weight loss in cancer patients is caused by loss of both adipose tissue and skeletal muscle mass, which is different from that caused by dietary restriction conserving lean body mass. Systemic and chronic inflammation also contributes to the symptoms in cancer cachexia. Inflammatory cytokines such as tumor necrosis factor-*α* (TNF-*α*), interleukin-1 (IL-1), interleukin-6 (IL-6), and interferon-*γ* (IFN-*γ*) are involved in the pathogenesis of cancer cachexia [4]. These cytokines have been reported to be involved in both anorexia and skeletal muscle loss [4]. Therefore, suppression of inflammation is thought to be a promising therapeutic approach for cancer cachexia [5–8].

Rikkunshito (RKT), a traditional Japanese herbal medicine (Kampo), is an extract of a mixture of* Atractylodis lanceae rhizoma, Ginseng radix, Pinellia tuber, Hoelen, Zizyphi fructus, Aurantii nobilis pericarpium, Glycyrrhizae radix*, and* Zingiberis rhizome*. Kampo prescriptions are manufactured in facilities complying with Japan's good manufacturing practice (GMP) and are fully integrated into the modern health care system in Japan [9]. RKT is prescribed for the treatment of functional dyspepsia, gastroesophageal reflux disease, dyspeptic symptoms in patients after gastrointestinal surgery, and chemotherapy-induced dyspepsia in cancer patients [10, 11]. It has been reported that RKT ameliorates anorexia, gastrointestinal dysmotility, and muscle wasting and prolongs survival in rats bearing ascites hepatoma cells (Yoshida AH-130) [12]. Additionally, RKT improves anorexia in a gastric cancer cachexia model [13] and in a bleomycin-induced acute lung injury model [14]. According to these reports, the action of RKT in anorexia would be mediated by stimulation of ghrelin secretion and/or potentiation of ghrelin signaling [12, 15, 16]. On the other hand, it has also been reported that RKT ameliorates bleomycin-induced acute lung injury by regulating lung inflammation independent of the ghrelin signaling systems [14]. Therefore, we first performed plasma metabolome analysis to explore other modes of action of RKT in cachexia and then evaluated changes in the key molecules identified. Metabolome analysis is the study of the characteristics and interactions of low molecular weight metabolites under a certain set of conditions, and changes in metabolite levels include the results of alterations of DNAs, RNAs, and proteins, because metabolites are located downstream of DNAs, RNAs, and proteins. Therefore, metabolome analysis is expected to provide valuable information which is more closely related to the phenotype of a cell, animal, or human and was applied in this study.

## 2. Materials and Methods

### 2.1. Animals

Male Wistar rats weighing 200–250 g were purchased from Japan CLEA (Tokyo, Japan) and housed in a regulated environment. Standard laboratory chow and water were available* ad libitum*. All experimental procedures were performed according to the Guidelines for the Care and Use of Laboratory Animals of Tsumura and Co. Ethical approval for the experimental procedures used in this study was obtained from the Laboratory Animal Committee of Tsumura and Co. All animal procedures were in accordance with the National Institutes of Health Guide for the Care and Use of Laboratory Animals.

### 2.2. Tumor Cell Inoculation

The rat ascites hepatoma AH-130 cell line was kindly donated by the Cell Resource Center for Biomedical Research, Institute of Development, Aging and Cancer, Tohoku University, Japan. A suspension of 10^8^ AH-130 tumor cells was prepared in 2 mL phosphate-buffered saline and injected intraperitoneally (i.p.) into rats.

### 2.3. Reagent Preparation

Rikkunshito, a traditional herbal medicine (TJ-43 spray-dried powder, Tsumura, Tokyo, Japan), was suspended in distilled water at a dose of 1,000 mg/kg for oral (p.o.) administration. The calorie content of the administered rikkunshito was negligible compared to dietary calorie intake. Potassium-D-glucarate (Sigma-Aldrich, St Louis, MO, USA) was dissolved in distilled water at doses of 2, 6, and 18 mmol/kg for p.o. administration.

### 2.4. Sample Preparation

Blood samples were collected into tubes containing aprotinin and EDTA-2Na then immediately transferred to fresh tubes and centrifuged for 3 min at 10,000 ×g. The collected plasma was stored at −80°C until use. To extract low molecular weight metabolites, 50 *μ*L of plasma was mixed with 250 *μ*L of a solvent mixture (MeOH : H_2_O : CHCl_3_ = 2.5 : 1 : 1) containing 10 *μ*L of 0.5 mg/mL 2-isopropylmalic acid (Sigma-Aldrich) dissolved in distilled water, and then the solution was shaken at 1,200 rpm for 30 minutes at 37°C before being centrifuged at 19,300 ×g for 5 minutes at 4°C. Two hundred and twenty-five microliters of the resulting supernatant was transferred to a clean tube and 200 *μ*L of distilled water was added. After mixing, the solution was centrifuged at 19,300 ×g for 5 minutes at 4°C, and 250 *μ*L of the supernatant was transferred to a clean tube and lyophilized using a freeze dryer (FDU-1200, Tokyo Rikakikai, Tokyo, Japan). For oximation, 80 *μ*L of 20 mg/mL methoxyamine hydrochloride (Sigma-Aldrich) dissolved in pyridine was mixed with a lyophilized sample before being shaken at 1,200 rpm for 90 minutes at 30°C. Next, 40 *μ*L of* N*-methyl-*N*-trimethylsilyl-trifluoroacetamide (MSTFA) (GL Science, Tokyo, Japan) were added for derivatization and the mixture was incubated at 1,200 rpm for 30 minutes at 37°C. The mixture was then centrifuged at 19,300 ×g for 5 minutes and the resulting supernatant was subjected to measurement by GC/MS.

### 2.5. GC/MS Analysis

The GC/MS measurements were performed using a GCMS-QP2010 Ultra (Shimadzu, Kyoto, Japan) with a fused silica capillary column (CP-SIL 8 CB low bleeds/MS; inner diameter: 30 m × 0.25 mm, film thickness: 0.25 *μ*m; Agilent Co., Palo Alto, CA, USA), and data analysis for the semiquantitative evaluation was carried out according to the method described in previous reports [17, 18].

### 2.6. Determination of Glucarate Content in Plasma and RKT

Glucarate content was measured as described in a previous report [19]. Bacterial enzyme solution was prepared from* Escherichia coli* (Migula; Castellani and Chalmers; ATCC 10536, ATCC, Manassas, VA, USA) by ammonium sulfate precipitation. Glucarate in a sample was converted to pyruvate by addition of bacterial enzyme solution. Then, converted pyruvate was assayed using lactate dehydrogenase and monitoring the absorbance of NAD^+^. Briefly, 105 *μ*L of assay buffer containing 67 mmol/L Tris-maleate, 67 mmol/L Mg_2_SO_4_, 0.5 mmol/L *β*-nicotinamide adenine dinucleotide (NADH) (Sigma-Aldrich), and 10 units lactate dehydrogenase (Oriental Yeast, Tokyo, Japan) were mixed with 40 *μ*L of each sample (plasma, 50 mg/mL RKT, or 50 mg/mL broccoli as a positive control) or potassium-D-glucarate as a standard in wells of a 96-well plate. The plate was incubated for 1 hour at room temperature to eliminate endogenous pyruvate in each sample. Then, 5 *μ*L of purified bacterial enzyme solution was added to each well, and it was incubated for another hour at room temperature. Absorbance at 340 nm caused by NAD^+^ production was measured at 0 and 60 min after addition of bacterial enzyme. The glucarate concentration in each sample was calculated using the standard samples.

### 2.7. Plasma *β*-Glucuronidase Assay

Plasma *β*-glucuronidase activity was determined by fluorometric assay according to a method reported previously [20]. Briefly, each plasma sample was mixed with 50 *μ*L of 0.1 mmol/L sodium acetate buffer (pH 4.6). After preincubation at 37°C for 5 min, 50 *μ*L of 0.5 mmol/L 4-methylumbelliferyl *β*-D-glucuronide was added to the plasma mixture and incubated at 37°C for 30 min. At the end of the incubation period, 150 *μ*L of 0.5 mol/L sodium carbonate was added to terminate the reaction. The concentration of 4-methylumbelliferone derived from 4-methylumbelliferyl *β*-D-glucuronide was quantified using FlexStation3 (Molecular Devices, Sunnyvale, CA, USA; Ex.: 360 nm, Em.: 465 nm). Plasma *β*-glucuronidase activity was calculated based on the level of 4-methylumbelliferone.

### 2.8. Real-Time PCR

Total RNA was extracted from samples using an RNeasy kit (Qiagen, Venlo, Netherlands), and DNA was removed from total RNA using RNase-Free DNase (Qiagen). Reverse transcription reactions were performed using a High-Capacity cDNA Reverse Transcription Kit (Life Technologies, Carlsbad, CA, USA). Expression levels of some targets were measured using TaqMan probes (Life Technologies) and those of others were measured using SYBR green reagent (Toyobo, Osaka, Japan). Primer sequences for SYBR green are shown in Table 1.

### 2.9. Analytical Assays

Levels of TNF-*α*, IFN-*γ*, and IL-10 in plasma were measured using an enzyme-linked immunosorbent assay (R&D Systems, Abingdon, UK) following the manufacturer's instructions.

### 2.10. Cytokine Induction in Rat Primary Splenocytes

Spleen mononuclear cells (SMC) were isolated from Wistar rats (7 weeks old). For preparation of SMC, erythrocytes were eliminated from a spleen cell suspension by hypotonic lysis in ammonium chloride and potassium chloride buffer. SMC were seeded in 24-well plates (3 × 10^5^ cells/well) in RPMI 1640 medium supplemented with 10% FBS, 100 units/mL penicillin and 100 *μ*g/mL streptomycin, 10 mmol/L HEPES, and 50 *μ*mol/L 2-mercaptoethanol and stimulated with 1 *μ*g/mL LPS for 4 h. Potassium-D-glucarate was simultaneously added to cultures at 10, 100, or 1,000 *μ*M. Expression levels of cytokines were measured according to the method for “real-time PCR” in Section 2.

### 2.11. Statistical Analysis

All data are expressed as mean ± SD or SE. The statistical significance of differences between 2 groups was evaluated by* F*-test analysis of variance, followed by Student's* t*-test. The statistical significance of differences between 3 or more groups was evaluated by Dunnett's test. A probability of less than 0.05 was considered statistically significant.

## 3. Results

### 3.1. Plasma Metabolome Analysis of RKT-Treated Tumor-Bearing Rats

**Figure 1:**
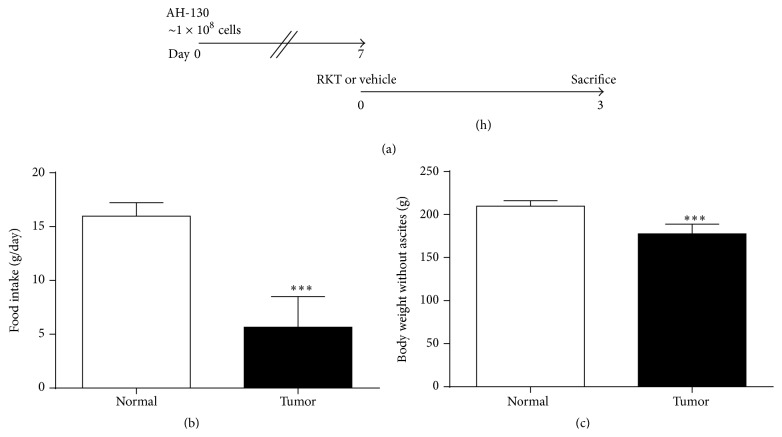
Experimental design of the preparation of plasma samples for metabolome analysis (a). Food intake (b) and body weight without ascites (c) in normal and tumor-bearing rats on day 7. Data represent mean ± SD (*n* = 30). ^*∗∗∗*^ *P* < 0.001 by unpaired Student's* t*-test versus controls.

To investigate the pharmacological mechanism of RKT in cachexia, plasma metabolome analysis was performed. The experimental design is shown in Figure 1(a). Wistar rats were inoculated with AH-130 ascites hepatoma at day 0. At day 7, significant reductions in food intake and body weight (without ascites) were observed in tumor-bearing rats, indicating that they had developed a cachectic phenotype (Figure 1(b)). At the same time point, RKT or its vehicle was orally administered to tumor-bearing rats and normal control rats, and plasma samples for metabolome analysis were collected 3 hours after administration. At least some changes in metabolites induced by various stimuli are known to occur in a short time, far faster than, for example, those in RNAs and proteins, which generally require several hours [21]. In contrast, RKT contains a number of components which exert their pharmacological activity only after metabolic conversion. It has been reported that glycyrrhetic acid, an important active substance which is converted from glycyrrhizin, could be detected 3 hours or more after administration of RKT in a clinical study [22]. Additionally, RKT improves gastrointestinal motility in tumor-bearing rats about 2 hours after administration [12]. Consequently, we decided to sample 3 hours after administration for metabolome analysis.

Low molecular weight metabolites were extracted from plasma, and metabolome analysis was performed by GC/MS as indicated in Section 2. A total of 110 metabolites were detected in the plasma from control and tumor-bearing rats (Supplementary Table 1 in Supplementary Material available online at http://dx.doi.org/10.1155/2015/871832). Among them, levels of 23 metabolites were significantly changed by the administration of RKT in tumor-bearing rats (Table 2). Levels of twenty metabolites increased in the RKT treatment group, and 3 metabolites decreased. In the normal control rats, 6 metabolites increased in the RKT treatment group, and 2 metabolites decreased. Glucarate, arabitol, and lauric acid were significantly changed by RKT treatment in both the tumor-bearing and control groups.

**Figure 2:**
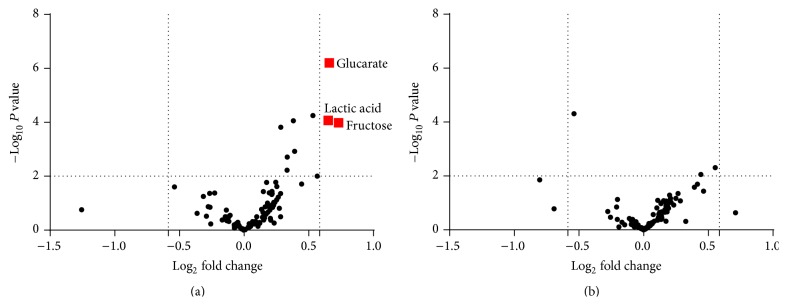
RKT treatment had a greater effect on plasma metabolites in tumor-bearing rats than in control rats. Volcano plots mapped by log_2_ mean value of RKT/vehicle at 180 min after treatment versus −log_10_ *P* value of* t*-test of RKT versus vehicle in tumor-bearing rats (a) and control rats (b). Red squares represent the metabolites which meet the criteria (*P* value < 0.01, fold change > 1.5 or <0.67).

Using volcano plots, which arranged the metabolites along axes of biological and statistical significance (fold change > 1.5 or <0.67, *P* value < 0.01), we found that RKT treatment seemed to have a greater effect on plasma metabolites in tumor-bearing rats compared to control rats (Figure 2). Three metabolites (glucarate, lactic acid, and fructose) showed a significant increase in tumor-bearing rats, while no metabolites met the criteria in normal rats. Since lactic acid and fructose were unchanged in the control group in response to RKT, it is possible that the increase of these metabolites in tumor-bearing rats may be caused not by administration of RKT but by inoculation of AH-130. In contrast, the level of glucarate was increased in both tumor-bearing and normal groups following RKT treatment and has previously been reported to have anticarcinogenic and antioxidative activities [23, 24]. Therefore, we further investigated whether glucarate had a beneficial effect on the symptoms of cancer cachexia.

### 3.2. Effect of Glucarate on Cachexia in Tumor-Bearing Rats

**Figure 3:**
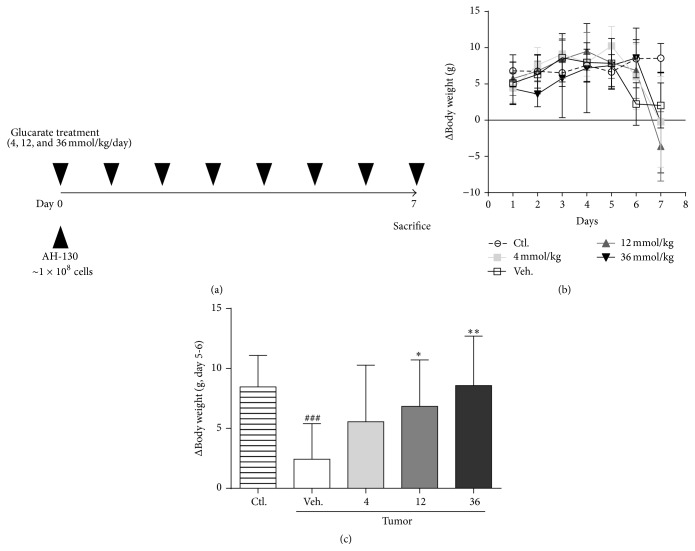
Glucarate treatment delayed the reduction in body weight gain in tumor-bearing rats. Experimental design of the evaluation of glucarate in a cachexia model rat (a). Daily body weight change over the experimental period (b) and body weight gain from day 5 to day 6 (c). Data represent mean ± SD (*n* = 10–12).  ^###^ *P* < 0.001 by unpaired Student's* t*-test versus control. ^*∗*^ *P* < 0.05; ^*∗∗*^ *P* < 0.01 by Dunnett's test versus vehicle control.

The experimental design is shown in Figure 3(a). AH-130 ascites hepatoma cells were inoculated at day 0, and then various doses of potassium-D-glucarate were orally administered for 7 days (4, 12, or 36 mmol/kg/day, divided into two doses). The rats were sacrificed on day 7. At the start of the study, 12 rats were prepared in each group. Some of the tumor-inoculated rats died or exhibited insufficient colonization of AH-130, and, as a result, the sample size of each group was 12, 11, 10, 10, and 11 in the control, tumor-vehicle, 4 mmol/kg, 12 mmol/kg, and 36 mmol/kg glucarate groups, respectively. The daily body weight change is shown in Figure 3(b). Although body weight gain in the 36 mmol/kg/day glucarate-treated tumor-bearing group was suppressed in the early period, it recovered after day 2. Body weight gain after day 5 was dramatically decreased in the tumor-bearing group without glucarate treatment. In contrast, glucarate treatment at all doses tested delayed the suppression of body weight gain (Figure 3(c)).

**Figure 4:**
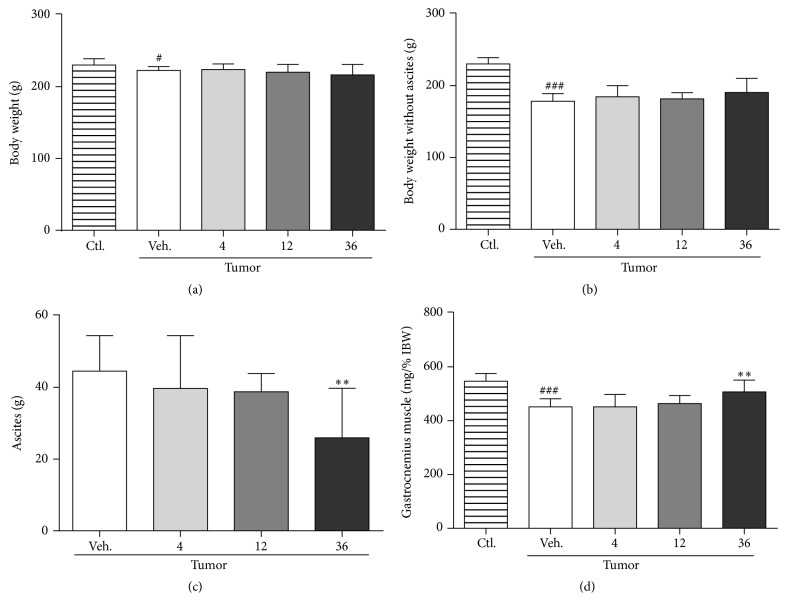
Glucarate treatment reduced ascites content and improved skeletal muscle atrophy. Effect of glucarate on whole body weight (a), body weight without ascites (b), content of ascites (c), and gastrocnemius muscle (d) at day 7. The weight of gastrocnemius muscle was normalized to initial body weight (IBW). Data represent mean ± SD (*n* = 10–12). ^#^ *P* < 0.05; ^###^ *P* < 0.001 by unpaired Student's* t*-test versus control. ^*∗∗*^ *P* < 0.01 by Dunnett's test versus vehicle control.

Although glucarate treatment did not improve body weight (with or without ascites) at day 7 (Figures 4(a) and 4(b)), 36 mmol/kg/day glucarate treatment significantly reduced the amount of ascites (Figure 4(c)) and ameliorated the loss of gastrocnemius muscle (Figure 4(d)). These results indicated that glucarate alone had beneficial effects on cancer cachexia.

**Figure 5:**
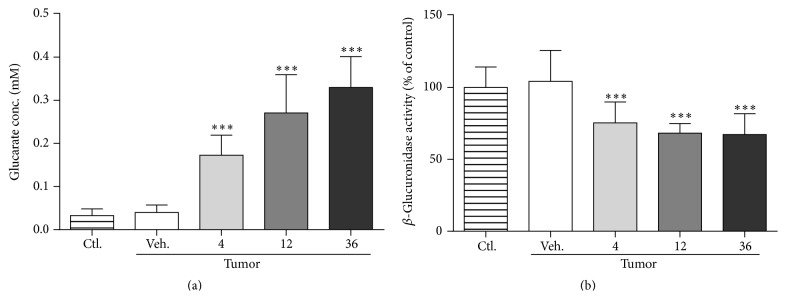
Glucarate treatment increased glucarate and suppressed *β*-glucuronidase activity in plasma. Effects of glucarate administration on plasma glucarate concentration (a) and *β*-glucuronidase activity (b). Inhibition of *β*-glucuronidase is likely to be caused by glucaro-1,4-lactone, spontaneously produced from glucarate in an aqueous environment. Data represent mean ± SD (*n* = 9–12). ^*∗*^ *P* < 0.05; ^*∗∗*^ *P* < 0.01; ^*∗∗∗*^ *P* < 0.001 by Dunnett's test versus vehicle control.

The glucarate concentration in plasma on day 7 was increased in a dose-dependent manner (Figure 5(a)). It has been reported that a portion of glucarate can be spontaneously converted to the potent *β*-glucuronidase inhibitor, glucaro-1,4-lactone [25]. In fact, we found that approximately 30% of *β*-glucuronidase activity was lost at all doses, suggesting that glucarate could be converted to glucaro-1,4-lactone (Figure 5(b)).

Since glucarate ameliorated gastrocnemius muscle atrophy, expression levels of enzymes or transcription factors involved in protein synthesis and degradation in the gastrocnemius muscle were measured. As shown in Table 3, mRNA expression levels of proteolytic enzymes (Atrogin-1 and MuRF-1) were increased in tumor-bearing rats. In contrast, the myogenic regulatory factor MyoD was reduced in the tumor-bearing group. However, glucarate did not change the expression levels of these enzymes. In addition, plasma thiobarbituric acid reactive substances, which are oxidative stress markers, were not changed by treatment with potassium-D-glucarate (data not shown).

**Figure 6:**
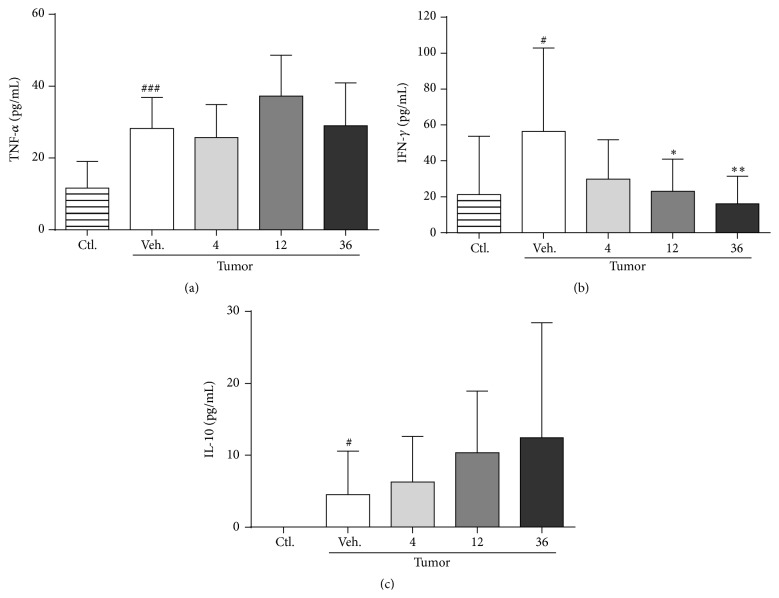
Glucarate improved the levels of plasma inflammatory cytokines in tumor-bearing rats. Effects of glucarate on the plasma levels of cytokines TNF-*α* (a), IFN-*γ* (b), and IL-10 (c). Data represent mean ± SD (*n* = 10–12). ^#^ *P* < 0.05; ^##^ *P* < 0.01; ^###^ *P* < 0.001 by unpaired Student's* t*-test versus control; ^*∗*^ *P* < 0.05; ^*∗∗*^ *P* < 0.01 by Dunnett's test versus vehicle control.

Finally, plasma cytokine levels were measured. Glucarate treatment was found to significantly reduce plasma IFN-*γ* level in a dose-dependent manner (Figure 6(b)). Of the other cytokines tested, glucarate tended to increase plasma IL-10 concentration (Figure 6(c)) and did not affect the level of TNF-*α* (Figure 6(a)).

### 3.3. Effect of Glucarate on Inflammatory Responses in Rat Splenocytes

**Figure 7:**
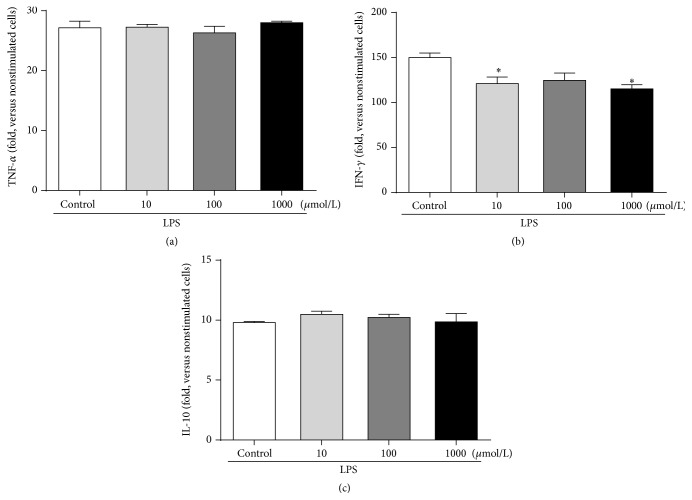
Glucarate inhibited the induction of IFN-*γ* expression in rat splenocytes. Effects of glucarate on expression levels in rat splenocytes of cytokines TNF-*α* (a), IFN-*γ* (b), and IL-10 (c). Rat splenocytes were stimulated with LPS for 4 h and total RNAs were prepared. Expression levels were measured by real-time PCR (SYBR green). Data represent mean ± SE (*n* = 3). ^*∗*^ *P* < 0.05 by Dunnett's test versus control.

Since glucarate treatment led to decreased plasma IFN-*γ*, we evaluated the effect of glucarate on inflammatory responses using primary splenocytes, because peripheral lymphocytes are believed to be a major source of IFN-*γ*. Rat primary splenocytes were treated with LPS and/or potassium-D-glucarate for 4 hours, and total RNA was isolated. The expression levels of cytokines were quantified by real-time PCR and the results are shown in Figure 7. As before, glucarate significantly reduced the expression level of IFN-*γ* (Figure 7(b)). This result indicates that glucarate directly affects splenocytes to regulate the plasma IFN-*γ* level. For nonstimulated splenocytes, glucarate gave no effect (data not shown).

### 3.4. Estimation of Glucarate Content in RKT

Glucarate is found in various fruits and vegetables, such as apple and broccoli [26]. The glucarate content in RKT extract was measured by a method involving enzymatic conversion of glucarate to pyruvate. The result showed that the glucarate content in RKT extract and in broccoli was 1.21 ± 0.42 and 0.18 ± 0.08 mg/g (mean ± SE, *n* = 3), respectively. These findings suggest that the increased glucarate levels observed in this study might be due to the glucarate content of RKT.

## 4. Discussion

In this study, using plasma metabolome analysis, we found that oral administration of RKT significantly increased plasma glucarate levels. Glucarate alone was shown to ameliorate cancer cachexia, reduce the amount of ascites, inhibit the loss of gastrocnemius muscle, and regulate the levels of inflammatory cytokines in plasma. The pharmacological activity of glucarate might be caused by a direct effect on lymphocytes. Since Kampo medicine contains many components and exerts pharmacological activity through its action on various targets, it is impossible to adequately explain the pharmacological mechanism of Kampo medicines by the activities of only one or a few components. Although extensive research on RKT has been performed, there are still many questions which remain to be answered such as the ghrelin-independent mechanism of RKT in a bleomycin-induced lung injury model [14]. Consequently, metabolome analysis is expected to be a powerful tool for the investigation of multicomponent based and multitargeted drugs such as Kampo medicines.

Metabolome analysis showed that levels of glucarate, arabitol, and lauric acid were significantly changed by RKT administration in both control and tumor-bearing groups. The pharmacological activity of arabitol is currently unknown. The level of lauric acid was significantly reduced in both groups. It has previously been reported that RKT reduces plasma free fatty acid levels as evaluated by meal challenge test in a clinical study [27], which was suggested to be caused by enhancing insulin secretion. Therefore, the reduction of lauric acid shown in this study might be caused by the same mechanism. It will be worthwhile to investigate the effect of RKT on other lipids such as different chain lengths of free fatty acid and triglycerides. Since lipid metabolism is reported to be altered in cancer cachexia [28, 29], RKT could affect cachexia by improving lipid metabolism.

Some metabolites were increased only in tumor-bearing rats. Among them, lactic acid and alanine are associated with pyruvate metabolism. It is well known that anaerobic metabolism is dominant in tumor cells (Warburg effect) [30] and Conde et al. reported that lactate and alanine production was increased in the tumor progression stage [31]. Furthermore, RKT contains sugars and amino acids which could convert to pyruvate after administration. Hence, the results obtained here are well aligned with these findings from preceding studies.

Glucarate was detected in RKT extract by an enzymatic method. In addition, glucarate esters have been identified in citrus peel [32], and RKT includes* Aurantii nobilis pericarpium* (*Citrus unshiu* peel). Kampo medicine contains various components which exert their pharmacological activity following their metabolic conversion. For example, glycyrrhizin is converted to glycyrrhetic acid in the process of absorption [33]. Glucarate esters could also be hydrolyzed by metabolic enzymes in the enteric microbiome, intestine, or liver. Hence, we suggest that the increase of plasma glucarate level following administration of RKT could be caused partly by the glucarate itself and partly by the hydrolysates of glucarate esters in RKT. However, there remains the possibility that RKT may induce endogenous glucarate.

Glucarate is a natural, nontoxic compound contained in fruits and vegetables [26]. It has been reported to block tumor initiation and progression [34, 35]. These activities are thought to be mediated by glucaro-1,4-lactone spontaneously produced from glucarate in aqueous solution. Glucaro-1,4-lactone is a potent *β*-glucuronidase inhibitor [25] and also has antioxidative activity [36]. The inhibition of *β*-glucuronidase enhances the clearance of carcinogens. Thus, glucarate has been demonstrated to have therapeutic effects in tumor models induced by carcinogens such as azoxymethane [35], 7,12-dimethylbenz[a]anthracene [37], and* N*-methyl-*N*-nitrosourea [34]. However, AH-130 ascites induced cachexia independent of the presence of toxic substances susceptible to glucuronidation. Therefore, it appears that detoxification is not involved in the pharmacological mechanism of glucarate in this cachexia model.

It should be noted that glucarate reduced the plasma level of IFN-*γ* in tumor-bearing rats (Figure 6). Additionally, we show for the first time that glucarate itself directly attenuates LPS-induced IFN-*γ* expression in rat primary splenocytes (Figure 7). AH-130 ascites is known to induce cachexia mainly via elevation of plasma TNF-*α* [38, 39]. IFN-*γ* is also reported to be a mediator of cancer cachexia [5]. Taking these facts into consideration, it is possible that glucarate could ameliorate the cachectic phenotype by regulating the expression of IFN-*γ*.

We demonstrate, by metabolome analysis, that RKT increases the plasma glucarate level and suppresses LPS-induced IFN-*γ* induction in splenocytes. IFN-*γ* is responsible for various diseases. In a bleomycin-treated mouse model, IFN-*γ* plays a proinflammatory or profibrotic role [40]. Therefore, RKT might ameliorate inflammatory responses by suppressing IFN-*γ* release in a bleomycin-induced model. Additionally, increased plasma glucarate leads to inhibition of *β*-glucuronidase activity through its conversion into glucaro-1,4-lactone. Therefore, it is expected that RKT could detoxify carcinogens, steroid hormones, and toxins in other diseases.

There are some limitations of this study. Our results show that glucarate reduces ascites weight (Figure 4(c)). Since the direct suppression of tumor growth by glucarate has been reported in some cases [41], the possibility that glucarate decreases the amount of ascites by suppressing the growth of AH-130 cells could not be excluded. Evaluation of the effect of glucarate on the cell number and volume of AH-130 would contribute to clarification of this point. Furthermore, glucarate ameliorated gastrocnemius muscle loss without affecting the expression levels of key modulators involved in protein synthesis and degradation processes (Figure 4(d), Table 3). It is possible that glucarate might affect the content of nonproteinaceous components in the gastrocnemius muscle, such as lipid and water. The analysis of composition and signaling pathways involved in metabolism of various biological materials would help to clarify this point. Finally, the effects of glucarate on normal animals have not yet been examined. We plan to carry out such investigations in future studies.

## 5. Conclusion

In summary, we performed plasma metabolome analysis and identified a valuable candidate molecule, glucarate, which is likely to be responsible for the anti-inflammatory activity of RKT. This method of metabolome analysis will be useful for the investigation of pharmacology in Kampo medicines.

## Supplementary Material

Supplementary Table 1: Plasma metabolites detected in this study.

## Figures and Tables

**Table 1:** Primers used for expression analysis in rat splenocytes.

	Forward	Reverse
GAPDH	5′-CCTGGAGAAACCTGCCAAGTAT-3′	5′-CTCGGCCGCCTGCTT-3′
TNF-*α*	5′-CGCCACCGGCAAGGA-3′	5′-GACATTCCGGGATCCAGTGA-3′
IFN-*γ*	5′-CGCCGCGTCTTGGTTTT-3′	5′-GAGTGTGCCTTGGCAGTAACAG-3′
IL-10	5′-CCCAGAAATCAAGGAGCATTTG-3′	5′-CAGCTGTATCCAGAGGGTCTTCA-3′

**Table 2:** Metabolites significantly changed in plasma of tumor-bearing and normal rats in response to RKT treatment.

Compound name	Tumor		Normal	
	Fold change	*P* value	Fold change	*P* value
Glucarate	1.58	<0.0001	1.31	0.026
Alanine	1.45	<0.0001	0.97	0.66
Lactic acid	1.57	<0.0001	1.05	0.58
Adipic acid	1.30	<0.0001	1.05	0.54
Fructose	1.66	0.0001	0.87	0.074
Aspartic acid	1.22	0.0002	1.01	0.84
Pyruvate + oxalacetic acid	1.31	0.0012	1.10	0.23
Oxalate	1.26	0.0019	1.64	0.23
Tryptophan	1.26	0.0059	0.86	0.14
Arabitol	1.48	0.010	1.47	0.0049
Fumaric acid	1.19	0.017	1.04	0.70
Lysine	1.13	0.017	1.07	0.15
Proline	1.36	0.019	0.96	0.75
Phenylalanine	1.19	0.024	0.99	0.94
*N*-Acetyl-L-glutamate	0.69	0.025	0.94	0.47
Phosphate	1.16	0.036	1.11	0.085
Glucono-1,4-lactone	1.11	0.037	1.13	0.096
Allantoin	1.14	0.041	1.03	0.58
Lauric acid	0.85	0.042	0.69	<0.0001
Cysteine + cystine	0.83	0.043	0.99	0.91
4-Hydroxyphenylacetic acid	1.22	0.044	1.03	0.79
Glucose	1.16	0.044	1.11	0.083
Urea	1.16	0.048	1.10	0.11

Each value represents the fold induction of the peak intensity in RKT-treated rats (*n* = 15) against that of vehicle-treated controls (*n* = 15). *P* values were calculated by unpaired Student's *t*-test.

**Table 3:** Expression levels of enzymes involved in protein synthesis and degradation in the gastrocnemius muscle.

(% of control)	Control	Vehicle	4 mmol/kg/d	12 mmol/kg/d	36 mmol/kg/d
Atrogin-1	100 ± 42	651 ± 321^###^	1135 ± 605	1133 ± 574	964 ± 889
MuRF-1	100 ± 31	364 ± 281^##^	552 ± 523	397 ± 390	545 ± 477
MyoD	100 ± 18	42 ± 19^###^	39 ± 31	33 ± 14	49 ± 41
Myogenin	100 ± 36	100 ± 56	102 ± 38	94 ± 60	82 ± 47

Data represent mean ± SD (*n* = 10–12). ^##^ *P* < 0.01; ^###^ *P* < 0.001 by unpaired Student's *t*-test versus control.
