# Supplementary material for: Rikkunshito Ameliorates Cancer Cachexia Partly through Elevation of Glucarate in Plasma
Source: Evid Based Complement Alternat Med. 2015 Sep 15;2015:871832. doi: 10.1155/2015/871832 (PMC4586964; doi:10.1155/2015/871832)
Supplement: Supplementary file 1 — Supplementary Table 1: Plasma metabolites detected in this study. [file 871832.f1.pdf]

Supplementary Table 1 Plasma metabolites detected in this study

Compound name	Tumor		Normal	
	Fold change	*P* value	Fold change	*P* value
Glucarate	1.58	< 0.0001	1.31	0.026
Alanine	1.45	< 0.0001	0.97	0.66
Lactic acid	1.57	< 0.0001	1.05	0.58
Adipic acid	1.30	< 0.0001	1.05	0.54
Fructose	1.66	0.0001	0.87	0.074
Aspartic acid	1.22	0.0002	1.01	0.84
Pyruvate + Oxalacetic acid	1.31	0.0012	1.10	0.23
Oxalate	1.26	0.0019	1.64	0.23
Tryptophan	1.26	0.0059	0.86	0.14
Arabitol	1.48	0.010	1.47	0.0049
Fumaric acid	1.19	0.017	1.04	0.70
Lysine	1.13	0.017	1.07	0.15
Proline	1.36	0.019	0.96	0.75
Phenylalanine	1.19	0.024	0.99	0.94
N-Acetyl-L-glutamate	0.69	0.025	0.94	0.47
Phosphate	1.16	0.036	1.11	0.085

Gulcono-1,4-lactone	1.11	0.037	1.13	0.096
Allantoin	1.14	0.041	1.03	0.58
Lauric acid	0.85	0.042	0.69	< 0.0001
Cysteine + cystine	0.83	0.043	0.99	0.91
4-Hydroxyphenylacetic acid	1.22	0.044	1.03	0.79
Glucose	1.16	0.044	1.11	0.083
Urea	1.16	0.048	1.10	0.11
3-Hydroxy-butyrate	0.80	0.056	1.00	1.00
Acetylsalicylic acid	1.20	0.060	1.15	0.05
N-FormylGlycine	1.19	0.076	1.17	0.12
Thymine	1.19	0.076	1.14	0.13
Prolinamide	1.19	0.076	1.14	0.13
Galactose	1.19	0.076	1.14	0.13
Dopa	1.19	0.076	1.14	0.13
N-α-acetyl-L-lysine	1.19	0.076	0.88	0.77
Raffinose	1.19	0.076	1.08	0.43
Uridine	1.18	0.093	1.13	0.21
Sucrose	1.17	0.093	1.10	0.31
Inositol	1.13	0.099	1.15	0.07
Putrescine	1.17	0.101	1.09	0.37

Threitol	1.16	0.114	1.12	0.22
Homoserine	1.17	0.119	1.10	0.31
Glutamic acid	1.14	0.127	1.04	0.56
Citrulline	1.14	0.134	1.10	0.26
Glutamine	0.83	0.136	1.13	0.09
Methionine	1.12	0.136	0.98	0.81
Nonanoic acid	0.83	0.141	0.97	0.72
4-Hydroxymandelate	1.16	0.144	1.13	0.13
5-Aminovaleric acid	1.21	0.156	1.38	0.04
1-Methyl histidine	1.10	0.169	1.05	0.27
Taurine	0.42	0.175	0.57	0.01
Homocysteine	1.15	0.180	1.14	0.13
Ornithine	0.91	0.181	1.13	0.18
Heptadecanoate	1.14	0.190	1.12	0.19
Elaidic acid	1.14	0.219	1.01	0.86
Malic acid	1.11	0.220	1.04	0.57
Shikimic acid	0.78	0.238	1.13	0.21
3-Hydroxyisovaleric acid	1.13	0.259	1.09	0.37
Glutaric acid	1.13	0.259	1.09	0.37
Pyrogallol	1.13	0.259	1.09	0.37

Glycerol-2-phosphate	1.13	0.259	1.09	0.37
2-Deoxy-D-glucose	1.13	0.259	1.09	0.37
Allose	1.13	0.259	1.15	0.09
Serotonin	1.13	0.259	1.16	0.10
Aconitate	0.93	0.282	1.10	0.21
Ribose	0.82	0.304	0.83	0.21
meso-Erythritol	1.22	0.317	0.90	0.65
Valine	1.07	0.320	0.97	0.76
Hippurate	0.90	0.321	1.36	0.01
O-Phosphoethanolamine	0.92	0.321	0.93	0.38
Xylitol	0.91	0.325	1.01	0.92
Ketoisoleucine	1.10	0.333	1.03	0.76
Cystathionine	1.11	0.354	1.12	0.18
Serine	1.15	0.367	0.94	0.62
Xylose	0.91	0.398	1.33	0.02
trans-4-Hydroxy-L-proline	1.11	0.423	1.06	0.47
S-Benzyl-L-cysteine	0.89	0.423	0.87	0.41
Glycolic acid	1.15	0.429	1.13	0.48
1-Hexadecanol	0.91	0.445	1.08	0.41
Palmitoleate	0.92	0.474	0.95	0.66

Kynurenine	1.06	0.478	1.02	0.80
Citric acid + isocitric acid	1.05	0.481	1.08	0.08
n-Caprylic acid	1.07	0.513	1.10	0.37
2-Aminoethanol	0.97	0.514	1.10	0.10
HydroxyButyrate	1.09	0.522	0.99	0.89
Histidine	1.18	0.543	1.25	0.48
Cysteine sulfonic acid	1.09	0.556	0.89	0.52
β-Alanine	1.04	0.559	1.10	0.22
Mannose	0.96	0.567	1.01	0.86
Histidine	0.96	0.573	0.94	0.41
1,5-Anhydro-D-glucitol	1.03	0.577	0.99	0.83
Uracil	0.97	0.583	1.02	0.73
Tyrosine	1.06	0.589	1.04	0.60
Galactosamine	0.84	0.590	0.62	0.17
Cysteic acid	0.95	0.635	1.04	0.59
Glucuronate	0.96	0.651	1.08	0.44
Succinic acid (or aldehyde)	0.97	0.669	1.08	0.26
Threonine	1.03	0.705	0.97	0.83
Ribitol	0.95	0.706	1.01	0.96
Glycine	0.97	0.711	0.96	0.50

Creatinine	1.08	0.716	1.08	0.34
N-Acetyl-L-glutamine	1.04	0.719	1.00	0.98
2-Aminobutyric acid	1.03	0.757	1.09	0.28
Asparagine	1.02	0.776	1.09	0.25
Ribulose	1.03	0.776	1.19	0.07
Uric acid	1.02	0.803	1.04	0.63
Benzen-1,4-dicarboxylic acid	0.98	0.817	1.14	0.16
Benzoic acid	0.95	0.845	0.84	0.34
Arabinose	0.99	0.885	1.22	0.08
Glyceric acid	0.99	0.887	1.09	0.23
2-Aminoisobutyrate	1.01	0.940	1.20	0.04
Cytosine	1.01	0.956	1.10	0.41
Phthalic acid	1.00	0.986	0.95	0.76
Glycerol	1.00	0.992	1.01	0.87
